# The impact of anchor characteristics on consumers’ willingness to pay a premium for food—an empirical study

**DOI:** 10.3389/fnut.2023.1240503

**Published:** 2023-09-04

**Authors:** Zhou Maojie

**Affiliations:** College of Tourism & Landscape Architecture, Guilin University of Technology, Guilin, China

**Keywords:** anchor characteristics, perceived value, premium purchase, limited-time, limited-quantity

## Abstract

In the food industry space, Netflix foods have exploded onto the Internet on the back of social media and many consumers are paying a premium for them. So what are the motives that may inspire consumers’ willingness to pay premium? In this paper, from the perspective of anchor, an external cue, a questionnaire survey was conducted with 275 respondents and analyzed using SPSS software. The results show that anchor characteristics (interactivity, professionalism and popularity) can influence consumers’ perceived value and increase their premium purchase intention. Perceived value mediates the relationship between anchor characteristics and willingness to pay a premium. Limited-time limited-quantity positively moderated the relationship between perceived value and premium purchase intention. The results reveal the key role of anchors in consumers’ decision-making process of buying Netflix food at a premium, and provide a theoretical basis for enterprises to select and cultivate anchors for product promotion.

## 1. Introduction

With the development of social media, the development pattern of the live broadcasting industry has gradually stabilized, and the integration of the netroots economy and social media has also shown strong resilience, and has been deeply integrated into the social production and life (1). In the field of food industry, Netflix food has become a newcomer in the food industry by relying on social media to explode all over the Internet (2). These foods often have unique packaging, ingredients and taste, and are welcomed and sought after by consumers. At the same time, along with the change of public consumption concepts and upgrading of consumer demand, consumers are willing to pay a premium for their favorite products (3). For Netflix food, even if the price is often higher than the actual value, it still attracts most consumers to pay for it (4). What makes consumers willing to pay a premium? Previous studies have found that consumers’ motivation to pay a premium is influenced by psychological factors (5). For example, consumers are willing to pay a premium for high-quality products due to product safety motives; consumers with a preference for place of origin are more likely to pay a premium for products with geographic landmarks (6). In addition, the external environment also influences consumers’ premium purchase decisions. For special products such as Netflix food, it is more difficult for consumers to understand their special product attributes through features such as product appearance, which prompts them to rely more on the live (external) environment to make purchasing judgments (7).

In the live shopping process, the anchor, as an opinion leader, plays the role of a bridge between the product and the consumer, which largely influences the consumer’s decision-making behavior (8). Therefore, enterprises pay more attention to the training of the anchor team, according to the target audience of the product or service to choose to match the different characteristics of the anchor to promote, in order to attract more attention from consumers (9). Take “EASTBUY” as an example, the agricultural products sold in this live broadcast are of good quality and higher price, and the anchor Dong Yuhui sold 320 million in 1 week with his own efforts. The anchor is extremely professional and knowledgeable, for the audience to create a “knowledge + entertainment + selling” of the new consumer experience and the high-priced agricultural products were purchased by consumers at a premium (10). Does the characteristics of the anchor affect consumers’ willingness to purchase food at a premium? Previous scholars have not focused on premium-priced products, although they have confirmed that anchor characteristics have an impact on consumers’ willingness to purchase (11). And, while previous studies have found that consumers are willing to pay a premium for foods with labels such as “green” and “organic,” or are influenced by personal factors (e.g., personal consumption preferences, personal consumption levels, etc.) to purchase high-priced foods (12), however, it has not focused on the role of the anchor as an external cue. Therefore, this paper attempts to explore the impact of anchor characteristics on consumers’ willingness to purchase food at a premium, starting with the external environment as a key factor influencing consumers’ willingness to pay at a premium. Furthermore, it has been suggested that e-commerce anchor characteristics influence consumers’ value perceptions first and then online purchase intentions (13). For food products, consumers rely on opinion leaders to make decisions, and when anchors are more interactive and professional, they enhance consumers’ trust and value perceptions of products. Meanwhile, product safety issues are frequent in the food sector, and buying products recommended by high-popularity anchors is more secure and affects consumers’ perceived value (14). Therefore, this paper will explore the mediating role of perceived value. Considering that companies often use limited-time limited-quantity marketing stimuli in product promotion, for example, the brand will set a specific time period for the sale of Netflix or limit the number of Netflix available to inspire a sense of urgency and scarcity among consumers. This sense of scarcity may have an impact on consumers’ perceived value (15), which in turn affects their purchasing decisions, so this paper introduces limited-time limited-quantity as a moderating variable.

Given the situational context of Netflix food in the social media era, it is necessary to investigate and fully understand consumer responses to Netflix food. And previous studies have not focused on the impact of the important role of the anchor, an external cue, on consumer premium payment in the online environment. Therefore, this study will explore the impact of anchor characteristics (interactivity, professionalism, and popularity) on consumers’ premium purchase intention, and further identify the intrinsic mechanism of action and boundary conditions of this process, so as to provide valuable insights for companies to choose the right anchors.

## 2. Literature review and research hypotheses

### 2.1. The SOR theory

The “Stimulus-Organic-Response” (SOR) model in marketing suggests that various external stimuli influence consumers’ physiological and psychological behavior by affecting their purchase behavior (16). In recent years, this theory has been widely applied to the study of online consumer behavior, and many scholars have explored the factors influencing consumers’ purchase intention from different perspectives based on this theory. Using the SOR theory, some scholars point out that the interactivity, professionalism and charisma of e-commerce anchors significantly affect consumers’ purchase intention based on the characteristics of e-commerce anchors’ opinion leaders (5). Based on the SOR model, other scholars have analyzed the influence mechanism of Internet Word of Mouth (IWOM) on college students’ willingness to purchase online (17). Based on the SOR model, some scholars have explored the influence of online reviews on consumers’ impulse purchase decisions (18). Given the importance of the SOR model in explaining the relationship between external factors and consumer responses, this paper designs a research framework on how anchors influence consumers to make food premium purchase decisions, investigates anchor characteristics (interactivity, professionalism, and popularity) as external stimuli, reflects state changes in emotion and cognition through perceived value, and further extends the SOR model by adding the moderating variable of limited-time limited-quantity.

### 2.2. Food premium payment

The issue of consumers’ willingness to pay has become a hot topic of academic attention in recent years. A review of previous studies reveals that most scholars have explored the motivations and reasons for paying a food premium from the perspective of the food itself or consumers’ personal preferences. Lang and Rodrigues (19) suggest that consumers are willing to pay a premium for quality food from an environmental and health perspective. Consumers are willing to pay a premium for foods with labels such as “organic” and “green” than for non-certified foods (20). At the same time, as environmental issues become more prominent and the concept of green and low-carbon is strengthened, the public is also willing to pay a premium for food products with a lower carbon footprint (21). In addition, some external factors may also influence consumers’ willingness to pay a premium. Huo et al. (22) found through an empirical study that credibility attributes stimulate consumer attitudes and increase consumers’ willingness to pay a premium for food products. Consumers prefer to pay a premium for packaged products over unpackaged product (23). It has also been noted that consumers have different attitudes toward paying a premium depending on the marketing channel (24). Table 1 summarizes articles that use external cues as a research perspective on how external factors affect consumers’ willingness to pay premiums.

**Table 1:** Studies of the effect of external factors on consumers’ willingness to purchase at premium prices.

References	Independent variable	Conclusion
Huo et al. (22)	Credence attributes (food safety, eco-friendliness)	Credence attributes stimulate consumers’ attitudes and increase consumers’ willingness to pay a premium (WTPP). Utilitarian attitudes and hedonistic attitudes play a partially mediating role in the relationship between credence attributes and WTPP. Uncertainty negatively moderates the role between utilitarian attitudes and WTPP, while it positively moderates the role between hedonistic attitudes and WTPP
Trivedi et al. (25)	Toxic-free food products	Both environmental concern and social media influence millennials’ attitude and purchase intention (PI). The PI so formed, in turn, has both a significant and positive influence on the WTPP for toxic-free food products
Zheng et al. (26)	Advertising appeals (green vs. non-green)	Self-construal and green agricultural product advertising appeals interact to influence consumers’ willingness to pay a premium for green agricultural products. Green perceived value plays a full mediating role in this interactive effect
Yu et al. (27)	Comparative advertising	The study found the positive effect of comparative advertising (vs. non-comparative advertising) in organic food communication, which has a positive effect on consumers’ willingness to pay premium for organic food
Li et al. (28)	Visual design (atmospheric cues)	Perceived luxuriousness leads to customers’ inferences of high quality of the coffee and high self-congruity, thus increasing WTPP. Further, cosmopolitanism moderates the effect of perceived luxuriousness only via self-congruity, but not via perceived quality
Calvo-Porral et al. (29)	Celebrity endorsement	Consumers are most influenced in their food consumption behavior by the congruence between the celebrity endorsement and the product being recommended, and by the celebrity credibility. Interestingly, celebrity recommendations show a similar influencing pattern both for consumers’ food purchase intention and consumers’ willingness to pay a premium price for food
Our research	Anchor characteristics	The three dimensions of anchor characteristics positively affect consumers’ perceived value, and further promote consumers’ willingness to buy food at a premium. When the time-limited marketing stimulus is used in live broadcasting, the relationship between perceived value and premium purchase intention can be positively adjusted

And anchors, as opinion leaders in social media, can have a significant impact on consumer decision-making. When anchors recommend a certain product or service in social media, consumers tend to pay high attention to their promotions. Influenced by social recognition and group effect, consumers are willing to pay a higher price for the products recommended by anchors. Therefore, as an important external cue for users to make decisions in the social media environment, the influence of anchors on consumers’ willingness to pay a premium for food cannot be ignored, and this study focuses on anchors as an external influencing factor.

### 2.3. Anchor characteristics and willingness to pay a premium

Live streaming has the characteristics of immediacy, interactivity, and authenticity, and consumers cannot make purchase decisions simply through the display of product appearance when buying food online, making their decision making behavior more dependent on online anchors (30). In the live shopping scenario, the anchors actively interact with consumers by showing the products, introducing their functions, ingredients, and effects, and then contribute to their purchase behavior (31). Different anchors will show different characteristics in the process of live-streaming with products, reflecting the professional ability and social attributes of the anchors, which have a guiding effect on consumers’ purchasing decision behavior (32). Combining the characteristics of marketing models in the food industry, this paper summarizes three main characteristics of anchors, which are interactivity, professionalism, and popularity. Interactivity is a key factor that influences the willingness to purchase in the food product line (33). Good interaction can motivate potential customers to buy (34). Especially for some high-priced food products, which are often of higher quality in terms of raw materials, production process and nutritional value, and such information is not easily understood by consumers, when there is good interaction between anchors and consumers, it can reduce information asymmetry, enhance consumers’ shopping experience and increase the willingness to purchase at a premium (35). Professionalism refers to the familiarity of the anchor with the product and the information related to it. The more professional the anchor is, the more consumers trust the efficacy and value of the products he or she introduces (36). Wang et al. (37) who suggest that consumers tend to make purchase decisions through the recommendations of opinion leaders, and when the anchor is able to explain clearly the reason for the premium price of the food product, it will lead to consumers’ purchase behavior. Popularity refers to how well known the anchor is to the public. Anchors with higher visibility tend to have higher influence and credibility in the industry (38). In a time when product quality and safety are prominent issues, consumers expect to purchase products that are guaranteed, and anchors with higher popularity are generally able to provide better guarantees. In addition, consumers’ food consumption behavior is influenced by celebrity endorsement, and consumers are more willing to pay a premium for food recommended by celebrities. Highly popularity anchors have a celebrity effect, and their recommended products can receive attention and elicit a premium purchase desire (39). Based on this, this paper proposes the following hypothesis:

*H1*: anchor characteristics positively influence consumers' willingness to pay a premium for food;

*H1a*: the professionalism of anchors positively influences consumers' willingness to pay at a premium;

*H1b*: the interactivity of anchors positively influences consumers' willingness to pay at a premium;

*H1c*: the popularity of anchors positively influences consumers' willingness to pay at a premium.

### 2.4. Mediating role of perceived value

Perceived value is a subjective judgment and measurement of the actual value of a product or service based on consumers’ own feelings (40). As the opinion leader in the live broadcast, the anchor’s own characteristics are an important factor to attract consumers. Special products such as food are difficult to make purchase decisions through factors such as simple physical characteristics, and professional anchors can convey information about the efficacy and value of the products to consumers, increasing their knowledge of the products, reducing uncertainty in the shopping process, and enhancing consumers’ perceived value (41). Highly popularity anchors are admired and sought after by the public because of their image appeal and social influence, and consumers can achieve a homogeneous identity with the anchors by purchasing the products they recommend (42). At the same time, high prices often signal economic superiority and high social status, and premium-priced foods recommended by high- popularity anchors can enhance consumers’ perceived social value (43). Some scholars found through empirical studies that higher interactivity is associated with higher consumer satisfaction and perceived value (44). Consumers are able to obtain more information about the food and enhance their perception by interacting with the anchor in real time while watching the live broadcast.

Before making a purchase decision, consumers integrate a range of information about a product to make a judgment about its value. In general, the higher the perceived value, the stronger the purchase motivation (45). When consumers are able to have a strong perception of the value of the recommended food, they will pay more attention to the quality and effectiveness of the food or service and will not be overly concerned about the financial gain or loss, resulting in a premium purchase behavior (7). Previous studies have also pointed out that in the process of live shopping, when consumers receive the stimulus of the anchor and the product, they form cognitive and emotional responses, and ultimately produce impulse consumption-like behaviors such as premium purchases (46). The cognitive and emotional components of perceived value can play a mediating role in external stimuli and consumer behavioral responses, which rationally explains the psychological mechanism that causes the relationship between anchors and purchase intention (47). According to the halo effect, when consumers have a better experience during the live broadcast of the anchor, they will build some confidence in the food they recommend, which will influence their perceived attitude and thus purchase intention (48). Therefore, the following hypothesis is proposed:

*H2*: Consumer perceived value has a positive effect on willingness to pay at a premium.

*H3*: Perceived value mediates the relationship between anchor characteristics and willingness to pay at a premium;

*H3a*: perceived value mediates the relationship between anchor professionalism and willingness to pay at a premium;

*H3b*: perceived value mediates the relationship between anchor interactivity and willingness to pay at a premium;

*H3c*: perceived value mediates the relationship between anchor popularity and willingness to pay at a premium.

### 2.5. Moderating effect of limited-time limited-quantity marketing stimulus

Limited-time limited-quantity is the provision of a product or service within a specific time period or limited quantity. It has been transformed from the early limited sale due to the scarcity of raw materials and the inability to mass produce due to seasonal and geographical constraints to a marketing-oriented marketing stimulus with additional content and meanings, which is an important tool for corporate marketing activities (49). When consumers are confronted with such marketing stimuli, they develop a nervousness and scarcity mentality, a process that accelerates the search for and identification of information, at which point they rely more on the power of online opinion leaders, i.e., they rely more on anchors to make purchasing judgments (50). While companies mostly use limited-time limited-quantity as a promotional strategy to achieve revenue, this paper focuses on a sense of scarcity it creates, emphasizing restricted purchase to attract target consumers. Under the marketing stimulus of limited-time limited-quantity, consumers tend to disregard long-term benefits and will make irrational purchases in pursuit of immediate pleasure (51). The perceived value of a product is related to product availability, and the lower the product availability, the higher the perceived value of the consumer (52). For certain food products, which may be in short supply in a particular season due to factors such as weather, consumers’ perceived value of the food product and thus their willingness to purchase it at a premium will be enhanced when they are faced with a limited-time limited-quantity event of the food product introduced during the live broadcast. In addition, products with limited-time limited-quantity labels often symbolize uniqueness, stimulate consumers’ perceived value, and stimulate the desire to purchase when purchase is limited, resulting in purchase behavior (53). Therefore, the following hypothesis is proposed:

*H4*: The limited time limit positively moderates the relationship between perceived value and willingness to purchase at a premium.

**Figure 1:**
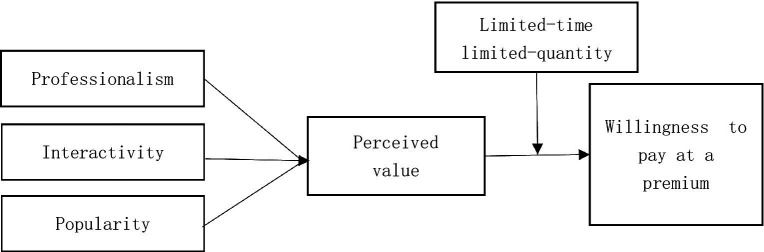
Theoretical model diagram.

Based on the above hypotheses, the theoretical model of this paper is shown in Figure 1.

## 3. Research design

### 3.1. Sample source and questionnaire design

This paper uses an online survey tool, Questionnaire Star, to distribute questionnaires and collect data. The survey was conducted from April to May 2023, and the sample was mainly selected from consumers who use social media. Participants used a “snowball” approach to share the questionnaire among their acquaintance networks, and those who could give positive feedback were rewarded with money. Two hundred and seventy-five valid questionnaires were returned.

In this paper, based on relevant studies, we designed a questionnaire on the influence of anchor characteristics on consumers’ willingness to purchase food products at a premium, taking into account the behavioral characteristics of food consumers. A five-level Likert scale was used to measure each variable. Among them, the measurement of anchor characteristics (professionalism, interactivity, and popularity) was mainly referred to Zhou et al.’s study; the measurement of perceived value was mainly referred to Ghali’s study; limited-time limited-quantity was measured by designing three questions according to Jang et al.’s study; and the willingness to purchase at a premium was measured by three question items from Tan et al. The specific measurement questions for each variable are shown in Table 2.

**Table 2:** Scale design and source.

Constructs	Item	Source
Professionalism	I think the anchor can introduce the information about the food clearly	Zhou et al. (54)
	I think the anchor can quickly answer viewers’ questions about food-related information	
	I think the slogan used by the anchor is in line with the facts of the food itself	
Interactivity	I think the anchor will respond positively to my questions during the broadcast	Wang et al. (55)
	I think I can understand the information about the food through the anchor	
	I think the anchor can communicate well with the audience	
Popularity	I think the anchor appears more frequently on the air	Chen et al. (56)
	I think the anchor has a certain reputation and fame	
	I think the anchor has some influence within the food marketing field	
Perceived value	I think the food recommended by the anchor has better quality	Ghali et al. (45)
	I think buying the food recommended by the anchor can improve my quality of life and enhance my personal image	
	I think I am more interested in the recommended food by watching the anchor live	
	I think the food recommended by the anchor has a high level of product safety	
Limited-time and limited-quantity	I think my desire to buy is enhanced when there are restrictions on the sale of food	Jang et al. (49)
	I think when a food is sold for a limited time, the food should be very good	
	I think when a food is sold for a limited time, this food is worth buying	
Willingness to purchase at a premium	I am willing to buy the food recommended by the anchor at a higher price than other channels	Tan et al. (7)
	I think the food recommended by the anchor is worth the higher price I paid for it	
	I would recommend to others to buy the food recommended by the anchor even though they have to pay a higher price	

### 3.2. Data collection

Table 3 shows the basic information of the sample of this survey. The results show that the gender composition of the valid sample is relatively balanced. In terms of the age composition of the sample, the majority of consumers are between 18 and 30 years old, and this age group is also the most used group of social media. In terms of education and income, the largest percentages are undergraduate and $5,000 to $6,000, respectively. This indicates that respondents have a relatively high level of education and income. Overall, the sample is well representative.

**Table 3:** Descriptive statistics.

Characteristic	Categorization	Frequency	Percent
Gender	Male	127	46.2%
	Female	148	53.8%
Age	18–25 years old	65	23.6%
	26–30 years old	81	29.4%
	31–40 years old	43	11.4%
	41–50 years old	33	12.0%
	51–60 years old	37	13.4%
	Over 60 years	18	10.0%
Education level	High school and below	39	14.2%
	Associate degree	56	20.4%
	Undergraduate	120	43.6%
	Master’s degree and higher	60	21.8%
Career	Government agencies, Institutions	43	15.6%
	Enterprise employee	54	19.6%
	Individual business	45	16.4%
	Farmer	43	15.6%
	Student	90	32.8%
Average monthly income	Below 5,000 yuan	50	18.2%
	5,000–6000 yuan	84	30.5%
	6,000–7000 yuan	64	23.3%
	7,000–8,000 yuan	43	15.6%
	More than 8,000 yuan	34	12.4%

## 4. Empirical analyses

### 4.1. Reliability and validity analysis

In this paper, SPSS and AMOS software were used to conduct validation factor analysis to test the reliability and validity of the sample data measuring anchor characteristics (professionalism, interactivity, and popularity), perceived value, limited-time limited-quantity, and willingness to pay at a premium, and the results of the analysis are shown in Tables 4, 5.

**Table 4:** Results of validation analysis.

Variable	Code	Cronbach’s alpha	CR	AVE	Standard load	S.E.
Professionalism	Q7	0.844	0.855	0.667	0.970	–
	Q8				0.743	0.035
	Q9				0.713	0.034
Interactivity	Q10	0.799	0.801	0.573	0.728	–
	Q11				0.790	0.099
	Q12				0.753	0.091
Popularity	Q13	0.801	0.801	0.573	0.753	–
	Q14				0.754	0.077
	Q15				0.763	0.081
Perceived value	Q16	0.846	0.849	0.585	0.721	–
	Q17				0.818	0.093
	Q18				0.701	0.088
	Q19				0.814	0.092
Limited-time limited-quantity	Q20	0.828	0.835	0.635	0.721	–
	Q21				0.972	0.099
	Q22				0.663	0.078
Willingness to pay at a premium	Q23	0.764	0.762	0.517	0.779	–
	Q24				0.715	0.076
	Q25				0.658	0.075

**Table 5:** Results of the discriminant validity test.

	A1	A2	A3	M1	M2	D
A1	0.817					
A2	0.649	0.757				
A3	0.734	0.580	0.757			
M1	0.664	0.570	0.670	0.765		
M2	0.700	0.532	0.620	0.659	0.797	
D	0.610	0.474	0.649	0.604	0.613	0.719

The letters in the table represent the following variables: A1, Professionalism; A2, Interactivity; A3, Popularity; M1, Perceived value; M2, Limited-time and limited-quantity; D, Willingness to pay at a premium.

From the results measured in Tables 4, 5, it can be seen that the Cronbach’s alpha coefficients of all variables in this paper exceed 0.7, and the combined reliability (CR) is greater than 0.7, indicating that the scales used in this study have good reliability. In addition, the standardized loadings coefficients in this study ranged from 0.658 to 0.972, the AVE were all greater than 0.5, and the square root of AVE were greater than the correlation coefficients between them and other constructs, indicating that the scale has good convergent validity and discriminant validity, and the reliability test passed (57).

### 4.2. Hypothesis testing

#### 4.2.1. Correlation analysis

In this paper, we first analyze the relationship between the variables using Pearson’s correlation coefficient. The mean, standard deviation and correlation coefficient of each variable are shown in Table 6. It was found that there were significant positive correlations between anchor characteristics (professionalism, interactivity and popularity), perceived value and willingness to pay at a premium. In addition, there was no significant correlation between the other demographic variables and the study variables, ruling out the effect of demographic variables, which provided a basis for further analysis.

**Table 6:** Correlation analysis.

	Average value	Standard deviation	D	M	A1	A2	A3	gender	age	A4	career	income
D	2.896	0.810	1									
M	2.948	0.798	0.604^**^	1								
A1	2.863	0.846	0.649^**^	0.670^**^	1							
A2	2.950	0.796	0.474^**^	0.570^**^	0.580^**^	1						
A3	2.922	1.040	0.610^**^	0.664^**^	0.734^**^	0.649^**^	1					
Gender	2.359	0.501	0.057	0.041	0.003	0.031	0.031	1				
Age	2.986	0.986	0.021	0.092	0.032	0.025	0.039	0.023	1			
A4	2.633	0.924	0.016	0.016	0.080	0.041	0.045	0.054	0.028	1		
Career	2.987	1.384	0.033	0.068	0.034	0.058	0.010	0.089	0.291	0.085	1	
Income	2.873	1.471	0.064	0.070	0.048	0.021	0.056	0.005	0.279	0.051	0.263	1

The letters in the table represent the following variables: A1, Professionalism; A2, Interactivity; A3, Popularity; M, Perceived value; D, Willingness to pay at a premium; A4, Educational level; * p < 0.05, **p < 0.01, ***p < 0.001.

#### 4.2.2. Regression analysis

In this paper, we first regress the professionalism, interactivity and popularity of anchor characteristics as independent variables and the willingness to pay at a premium as dependent variables, and the results are shown in Table 7. The results show that professionalism (*t* = 6.327, *p* = 0.000) and popularity (*t* = 3.620, *p* = 0.000) have a positive effect on the willingness to pay at a premium, and H1a, H1c are confirmed, while interactivity (*t* = 1.008, *p* = 0.314) does not have an effect on the willingness to purchase at a premium.

**Table 7:** Regression analysis of anchor characteristics on willingness to pay at a premium.

	Non-standardized coefficient B	*t*	*p*	VIF
Professionalism	0.406	6.327	0.000**	2.261
Interactivity	0.061	1.008	0.314	1.799
Popularity	0.202	3.620	0.000**	2.593

*p < 0.05, **p < 0.01, ***p < 0.001.

Next, the regression analysis of perceived value on willingness to pay at a premium was conducted, and it was found that perceived value (*t* = 12.531, *p* = 0.000) positively influences willingness to pay at a premium, and H2 was confirmed.

#### 4.2.3. Mediation effect test

In this paper, we use model 4 in the process plug-in of SPSS to test for mediating effects (58), We chose the Bootstrap test and set the number of repetitions to 5,000 and the confidence interval to 95% for testing. The results of the study are shown in Table 8.

**Table 8:** Results of mediation effect test.

Path	Direct effect		Indirect effect	
	β	Bootstrap 95% Cl	β	Bootstrap 95% Cl
Professionalism→M→D	0.424	0.194 ~ 0.453	0.338	0.029 ~ 0.148
Interactivity→M→D	0.060	−0.101 ~ 0.136	0.017	0.010 ~ 0.087
Popularity→M→D	0.260	0.038 ~ 0.258	0.190	0.019 ~ 0.139

M denotes perceived value, D denotes willingness to pay at a premium.

The bootstrap 95% confidence intervals for both the indirect effect [0.029, 0.148] and the direct effect [0.194, 0.453] of perceived value in path 1 do not contain 0, suggesting that perceived value partially mediates the relationship between anchor professionalism and willingness to pay at a premium. The bootstrap 95% confidence intervals for the indirect effect [0.010, 0.087] and the direct effect [−0.101, 0.136] of perceived value in path 2 contain 0, indicating that perceived value fully mediates the relationship between anchor interactivity and willingness to pay at a premium. The bootstrap 95% confidence intervals for both the indirect effect [0.019, 0.139] and the direct effect [0.038, 0.258] of perceived value in path 3 do not contain 0, indicating that perceived value partially mediates the relationship between anchor popularity and willingness to pay at a premium. Therefore, all hypotheses of H3 hold.

#### 4.2.4. Moderating effect test

In this paper, we base on the model 14 proposed by Hayes (59) for the moderated mediation test. The tests for mediating effects with moderation are shown in Table 9. When the independent variable is anchor professionalism, the mediating effect of perceived value is insignificant at the low limited-time limited-quantity level (indirect effect = 0.041, Boot CI = [−0.018, 0.107]), while the mediating effect of perceived value is significant at the high limited-time limited-quantity level (indirect effect = 0.072, Boot CI = [0.014, 0.109]). The results indicate that there is a significant difference between the low and high limited-time limited-quantity groups in terms of whether premium purchase intentions are influenced through perceived value, with the mediating effect of perceived value being moderated by the limited-time limited-quantity marketing stimulus. Similarly, when the independent variable was anchor popularity/interactivity, the limited-time limited-quantity also strengthened the mediating effect of perceived value between anchor popularity/interactivity and willingness to pay at a premium, and hypothesis H4 was confirmed.

**Table 9:** Results of the test for moderating effect.

Independent variable	Intermediate variables	Level of adjustment variables	Effect	Boot CI
Professionalism	Perceived value	Low level	0.041	[−0.018, 0.107]
		High level	0.072	[0.014, 0.109]
Interactivity		Low level	0.021	[−0.009, 0.066]
		High level	0.038	[0.004, 0.081]
Popularity		Low level	0.027	[−0.012, 0.078]
		High level	0.047	[0.007, 0.019]

### 4.3. Summary

In this study, each hypothesis was tested by regression analysis, mediating effect test, and moderating effect test, and all the hypotheses held except hypothesis H1b. This indicates that in the social media era, anchors as opinion leaders largely influence consumers’ decision-making behavior. For premium food such as Netflix food, the professionalism and popularity of the anchor play an important role in influencing consumers’ premium purchase intention. Professional anchors can clearly convey all kinds of product attributes to consumers, and well satisfy consumers’ information demand. The higher the popularity of the anchor, the greater the stickiness of its fans, which can better form a group effect, and then stimulate the consumer’s willingness to buy at a premium. The anchor interactivity does not have a direct impact on consumers’ premium purchase intention. This may be due to the fact that for food products, consumers do not have high personalized demands and do not rely on highly interactive anchors to give targeted recommendations. In addition, perceived value plays a mediating role in the influence of anchor characteristics on consumers’ premium purchase intention. At the same time, the marketing stimulus of creating a sense of scarcity at the same time can stimulate consumers’ desire to buy and facilitate the sales of premium products.

## 5. Discussion

### 5.1. Theoretical significance

This paper focuses on the influence of anchors, an external factor, on consumers’ willingness to purchase food at a premium, broadening the scope of research in food-related fields. In the era of social media, anchors, as opinion leaders, largely influence consumers’ decision-making behavior. Although a large number of studies have emphasized the role of anchors in consumers’ purchasing behavior, they have not addressed the study of food products (60). And it is worth exploring whether the external cue of anchors plays a role in food and beverage as a popular area in live streaming (61). This paper confirms through empirical research that the professionalism and popularity of anchors can have an impact on consumers’ willingness to purchase food products at a premium. And the professionalism of the anchor has the greatest impact on consumers’ willingness to pay at a premium, which means that consumers pay more attention to the professional output of the anchor when purchasing premium food products (62). This process can effectively reduce the information gap, so that consumers can understand the source of the premium “label” and enhance their perceived value. The results of this study provide a new theoretical basis for companies to develop marketing strategies for premium foods and select appropriate anchors for promotion.

Second, this paper reveals the intrinsic psychological mechanisms of consumers in the process of purchasing premium foods and promotes the explanation of the relationship between anchor characteristics and willingness to pay at a premium. Although existing studies have emphasized the mediating role of perceived value between anchor characteristics and product purchase intentions without focusing on premium-priced products (63). At the same time, some scholars have also pointed out that the psychological factor of consumers’ perceived value will have an effect on premium purchase intention, but it has not been considered from the perspective of anchor characteristics (64). In conclusion, premium foods require consumers to pay higher prices compared to similar foods, and it has not been confirmed by scholars whether the mediating variable of perceived value also plays a role in the effect of anchor characteristics on consumers’ willingness to purchase at a premium (31). This study found that perceived value still plays a role in the effect of anchor characteristics on premium food purchases, which enriches the scope of research on perceived value as a mediating variable.

Finally, this paper enhances the explanatory power of the SOR model by adding limited time limit as a moderating variable of the model. The model explains that consumers’ willingness to pay premium prices for online food is driven and constrained by anchor characteristics, perceived value, and limited-time limited-quantity. The empirical study identifies the moderating role of the marketing stimulus of limited-time limited-quantity in the relationship between perceived value and willingness to pay premium, and refines the paths through which different characteristics of anchors influence consumers’ premium purchase behavior through perceived value (65). While most scholars have previously studied this marketing stimulus as a promotional strategy, for example, this paper focuses on the sense of scarcity created by limited-time limited-quantity (66). Product scarcity creates a positive impact that can increase the pleasure of consuming a product and promote consumers’ perceived value of that product. The current study finds that limited time limits strengthen the mediating role of perceived value between anchor characteristics and premium purchase intentions, expanding the boundaries of existing research on premium food purchases.

### 5.2. Management significance

First, companies need to develop a reasonable marketing strategy and choose the right anchor for product promotion. Specifically, the most critical thing is to consider the professionalism of the anchor. Due to the special nature of food products, the professional introduction of the anchor is needed to make consumers understand more clearly the relevant product attributes and characteristics and other information (67). In addition, try to choose high-profile anchors to promote related products. Netflix food is often popular among the public, and high-profile anchors who “match” them are often better able to play a good publicity effect, thus increasing consumers’ willingness to buy food at a premium.

Secondly, when promoting food products to consumers, companies should pay attention to the perceived value of consumers. For example, you can consider the design of eye-catching, beautiful, unique product packaging, incorporating trendy elements, highlighting the visual effects and other ways to enhance consumer perception of entertainment value. Nowadays, selling “emotion” has become one of the selling points of “Netflix” food, and actively creating a unique brand style can enhance consumers’ emotional value perception (68). In addition, food safety accidents are frequent, and companies should strengthen their brand image to ensure food safety and show that they attach great importance to food quality and safety, so as to enhance consumers’ perception of safety value.

Finally, companies should take appropriate marketing stimuli to increase consumers’ willingness to buy at a premium. Food products are often affected by seasonal and other factors only launched in a specific season, for this type of food, companies can adopt a limited-time limited-quantity marketing strategy to stimulate consumers’ desire to buy. Companies can also reduce the production of food products to make them in short supply, so as to achieve the purpose of maintaining high popularity means (69). In addition, if companies want to increase consumer stickiness to gain more profits, they can increase the restrictions on consumer status and other restrictions to improve consumer loyalty to the brand, and how to make the Netflix food long red is a problem worth thinking about for companies.

### 5.3. Limitations and prospects

First, in terms of research perspective, this paper only explores the impact of different anchor characteristics on consumers’ willingness to pay at a premium, while the interaction of different types of anchors, anchor types and product types often also has different effects on consumer behavior (70). Second, although this paper focuses on the moderating role of limited-time limited-quantity, it does not make a strict distinction between such marketing stimuli (71). And although both limited-time or limited-quantity scarcity marketing stimuli can have an impact on consumers’ purchase intentions, there may be differences in the degree of impact between these two stimuli (72), future research could focus on this difference for a deeper exploration. Finally, factors such as consumers’ personal preferences may affect the willingness to purchase at a premium, and future research could explore the underlying mechanisms and boundary conditions of consumers’ premium purchasing behavior more fully and rationally.

## Data availability statement

The original contributions presented in the study are included in the article/supplementary material, further inquiries can be directed to the corresponding author.

## Author contributions

The author confirms being the sole contributor of this work and has approved it for publication.

## Funding

Guangxi science and technology plan project (guikeAB23026053), Guangxi University Young and middle-aged teachers’ basic scientific research ability improvement project (2021KY1675), Guilin science and technology research and development project (20180102-2).

## Conflict of interest

The author declares that the research was conducted in the absence of any commercial or financial relationships that could be construed as a potential conflict of interest.

## Publisher’s note

All claims expressed in this article are solely those of the authors and do not necessarily represent those of their affiliated organizations, or those of the publisher, the editors and the reviewers. Any product that may be evaluated in this article, or claim that may be made by its manufacturer, is not guaranteed or endorsed by the publisher.
